# Cell Behavior and Complex Mechanical Properties of 3D Printed Cell‐Laden Alginate‐Gelatin Macroporous Mesostructures

**DOI:** 10.1002/mabi.202500204

**Published:** 2025-09-17

**Authors:** Nicoletta Murenu, Jessica Faber, Anahita Ahmadi Soufivand, Monika Buss, Natascha Schaefer, Silvia Budday

**Affiliations:** ^1^ Institute For Clinical Neurobiology University Hospital Würzburg Würzburg Germany; ^2^ Institute of Continuum Mechanics and Biomechanics FAU Erlangen‐Nürnberg Fürth Germany

**Keywords:** ALG‐GEL, bioprinting, cyclic compression‐tension, long‐term cell viability, pore size

## Abstract

Bioprinting involves additive manufacturing of materials containing living cells, known as bioinks, which are formulated from cytocompatible hydrogel precursors. The bioink's characteristics before, during, and after crosslinking are critical for its printability, structural resolution, shape fidelity, and cell viability. The mechanical properties of printed constructs can be strongly influenced by their macroporous mesostructure, including pore size, filament diameter, and layer height, and are crucial for the intended applications in tissue engineering or regenerative medicine. It is known that the mechanical properties of hydrogels influence cell performance, but in turn, cells can also alter the mechanical properties of bioprinted constructs, which remain poorly understood. To explore these interdependencies, we selected an alginate‐gelatin hydrogel (ALG‐GEL), due to its well‐known biocompatibility, combined with U87 cells and bioprinted three different multilayer macroporous mesostructures with varying porosity and filament diameter. We investigate how different macroporous mesostructures affect cells, how cells, in turn, influence mechanical properties, and whether the stability and mechanical properties of bioprinted macroporous mesostructures change over time. Our findings show that the bioprinted constructs are stable over the course of 14 days and highlight that cells can significantly influence their mechanical properties. This has important implications for biofabrication and tissue engineering applications.

## Graphical abstract

Macroporous mesostructures were bioprinted using ALG‐GEL bioink loaded with U87 cells. Their printability was not affected by the cells, and the constructs were stable over 14 days. Mechanical properties and viability of cells were analyzed at days 0, 1, and 14, showing that the macroporous mesostructure did not affect cellular viability. On the contrary, cells have had an impact on mechanical properties.

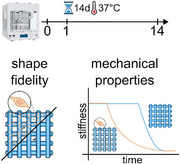

## 1 Introduction

Bioprinting surpasses traditional biofabrication methods by enabling the precise creation of complex, tissue‐like constructs [1, 2, 3]. Unlike conventional techniques, 3D bioprinting allows for the controlled deposition of cells and biomaterials in predefined patterns [4, 5]. The printed constructs must replicate the native tissue's stiffness and mechanical behavior to integrate effectively and support essential mechanical and cellular functions, such as growth and differentiation [6, 7, 8]. Moreover, stability under physiological forces is crucial, particularly for load‐bearing tissues like bone and cartilage [4, 9]. By optimizing mechanical properties, bioprinted constructs can achieve both load‐bearing capacity and biological functionality, ensuring their effectiveness in tissue engineering and regenerative medicine [10].

The mechanical properties of printed constructs can be strongly influenced by their macroporous mesostructure, including pore size, filament diameter, and layer height [4, 11, 12]. These structural characteristics are critical in determining load‐bearing patterns, nutrient delivery, and cellular activity [4, 13]. Despite their importance, the influence of different macroporous mesostructures has hardly been investigated due to challenges in maintaining structural integrity and shape fidelity during 3D bioprinting [14, 15]. Our recent study showed that the macroporous mesostructure affects the mechanical properties of cell‐free 3D bioprinted alginate‐gelatin constructs immediately after printing [16]. However, incorporating cells and evaluating mechanical properties at additional time points *in vitro* could further illuminate how macroporous mesostructures affect mechanical properties as well as cell behavior and, in turn, how cells influence the construct's mechanical properties over time.

A fundamental requirement for bioprinting tissue constructs is using a biocompatible and printable bioink [17, 18]. Various bioinks have been explored in the literature, including alginate, gelatin, hyaluronic acid, gelatin methacrylate (GelMA), and extracellular matrix‐based bioinks, applied either individually or in composite formulations [19, 20, 21]. Among these, a composite bioink made from gelatin and alginate has gained significant attention [22, 23, 24]. Gelatin, on the one hand, exhibits thermo‐gelling properties, enabling structures with high shape fidelity during printing [25]. Alginate, on the other hand, contributes crosslinking capabilities, improving the structural stability of the bioink [22]. Furthermore, alginate‐gelatin hydrogels are particularly advantageous because they are easy to prepare, provide a cell‐friendly environment, and support cellular growth and proliferation, making them a versatile choice for bioprinting applications.

In a previous study, we had characterized the mechanical properties of alginate‐gelatin (ALG‐GEL) macroporous mesostructures—printed in multilayer structures with different pore sizes, sample heights, and filament diameters [16]. Based on these findings, we now aim to explore how the incorporation of cells affects the mechanical properties of the printed structures. In addition, we assess the effect of the macroporous mesostructured hydrogel matrix on cellular behavior. Specifically, we seek to understand when and how the presence of cells influences the material's behavior, i.e., its stiffness, nonlinear elasticity, and overall structural integrity. For this purpose, we chose U87 cells, which are a well‐characterized glioblastoma cell line known for its robustness and high proliferative capacity [26]. Notably, this cell line has been utilized in various studies involving 3D bioprinting and different hydrogel matrices, demonstrating that it can tolerate the printing process well and maintain viability and functionality post‐printing [27, 28]. These characteristics make U87 cells particularly suitable for exploring how cellular interactions influence the mechanical properties of biofabricated macroporous mesostructures and vice versa. In addition, in several studies, it could be shown that neural stem and primary cells have successfully been used in the gelatin‐alginate systems for culturing [29, 30, 31]. In this study, we bioprint three different U87‐loaded types of macroporous mesostructures characterized by different pore sizes and thus different macroporosities. Samples were kept in culture for 14 days, monitoring stability of the construct, cell performance, and their influence on mechanical properties at days 0, 1, and 14.

## 2 Results and Discussion

**FIGURE 1:**
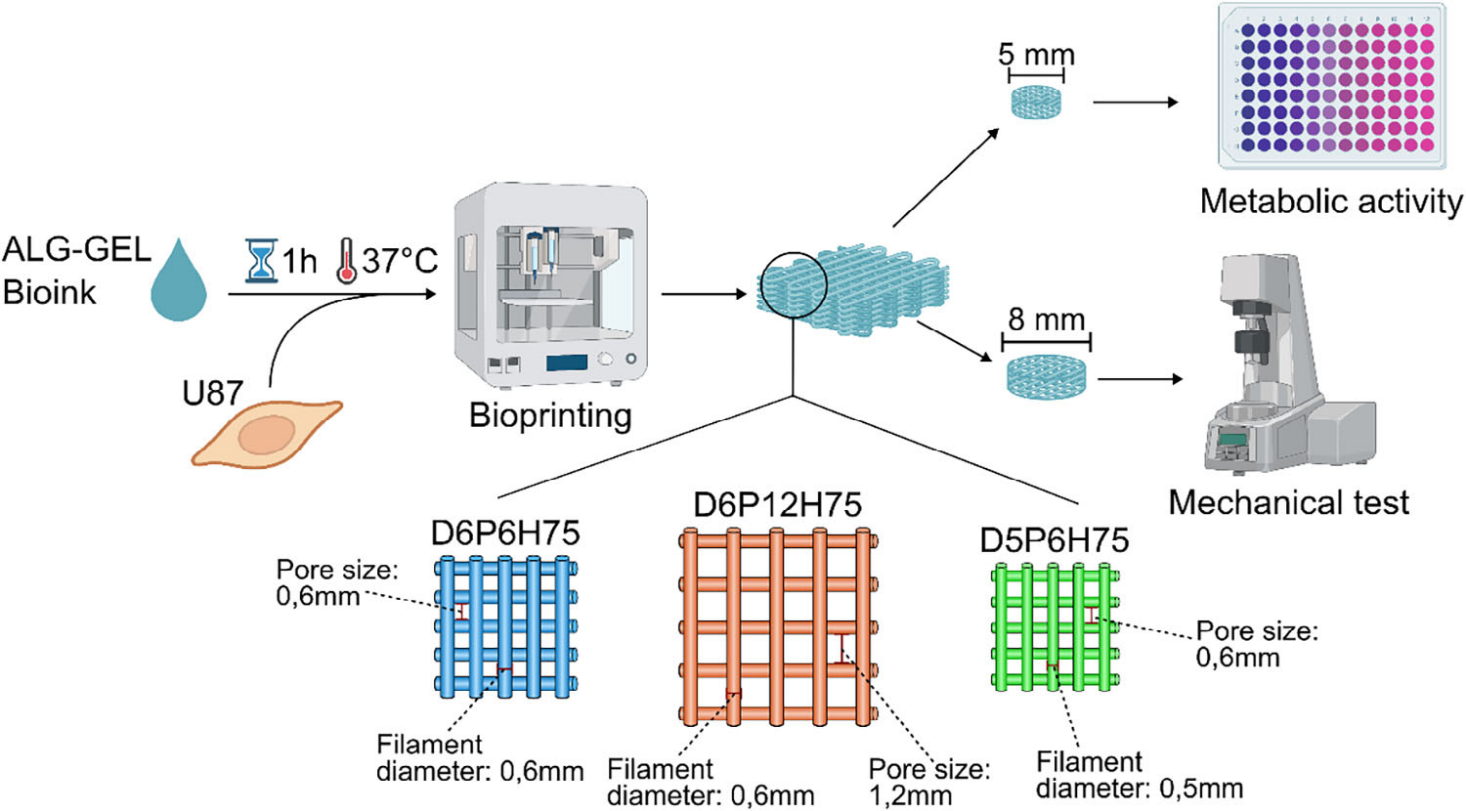
Schematic overview of experimental procedure. From left to right: U87 cells were loaded into the ALG‐GEL bioink and, after incubation, different constructs (bottom) were bioprinted (D6P6H75, D6P12H75, and D5P6H75). After printing (middle), cylinders of different diameters (5 or 8 mm) were punched. 5 mm cylinders were used for metabolic activity measurements (upper right), while 8 mm punches were mechanically analyzed (lower right). Created in BioRender. Schaefer, N. (2025) https://BioRender.com/md7wnbp.

Figure 1 shows a schematic overview of the study design. Based on our previous findings, we selected three specific macroporous mesostructures (Figure 1, bottom) for further investigation regarding the influence of the incorporation of cells, as these had shown clear differences in their mechanical properties without cells [16]. The selected macroporous mesostructures differ in their pore size and/or filament diameter. They are named DxPyH75, referring to a nominal filament diameter (Dx) and pore size (Py), and a layer height of 75% of the filament diameter (H75).

We utilize the human glioblastoma cell line, U87, well known to be a standard model for evaluating the biocompatibility of hydrogels. These cells tend to exhibit a good survival rate during the printing process [27], which makes them an appropriate choice for testing the mechanical and biological properties of printed cell‐laden macroporous mesostructures.

Initially, the hydrogel was printed into square‐shaped samples, from which cylindrical samples were extracted using two different sizes of punches (5 and 8 mm). For mechanical testing, 8 mm cylindrical samples were carefully obtained from the square samples using a surgical punch and mounted to the rheometer. In parallel, five 5 µm diameter samples were prepared for the analysis of metabolic activity in order to evaluate the cellular behavior within the hydrogel macroporous mesostructure (Figure 1, top).

### 2.1 Effect of Cells on Printability, Stability, and Cell Viability

**FIGURE 2:**
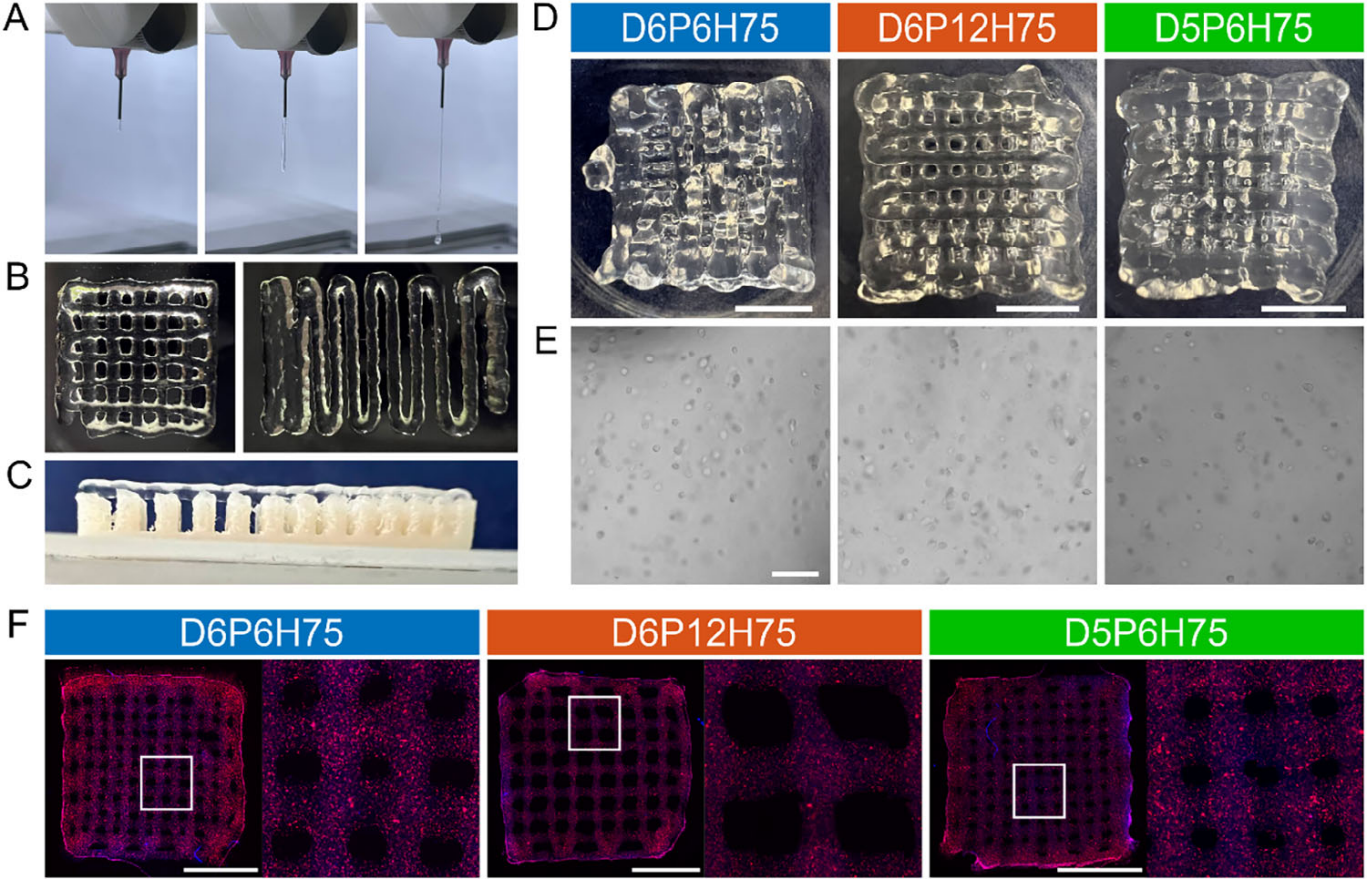
Printing cell‐laden 3D ALG‐GEL samples. All printing tests were conducted with a 600 µm needle, at 27°C, and under constant pressure of 90 kPa. (A) Extrusion filament. (B) Printing stability tests: two‐layer grid (left) and filament fusion (right). (C) Collapse test. (D) Representative pictures of cell‐laden bioprinted constructs with different macroporous mesostructures, D6P6H75, D6P12H75, and D5P6H75, right after bioprinting. Scale bar 5 mm. (E) Brightfield images of U87 cells embedded in the hydrogel after printing. Scale bar 100 µm. (F) Fluorescent tdTomato (red) transduced U87 cells embedded in the hydrogel. The left image shows the whole printed macroporous mesostructure, and the right image depicts the magnification of the marked area. Nuclei were stained with DAPI (blue). Scale bar 5 mm.

To ensure that the bioink loaded with cells was printable in a continuous and controlled way, we first investigated the uniformity of the extruded bioink (Figure 2A). This property is essential for precise and consistent printing. Ensuring uniformity helps to avoid needle clogging, filament breakage, and irregular filament formation, all of which can compromise the quality and accuracy of the final printed structure [32]. The bioink achieved a continuous and controlled flow under a constant pressure of 90 kPa at 27°C.

Proper gelation of the bioink was also assessed by printing two layers of crossed patterns (Figure 2B left) to calculate the P_r_ value as a measure of the bioink's printability. For a bioink with optimal gelation and flawless printed structures, the interconnected grid holes will form perfect squares, achieving a P_r_ value of 1. Since the ALG‐GEL bioink formulation achieved a P_r_ value of 0.88±0.01, it was considered sufficiently adequate to proceed. In addition, we performed the filament fusion test with a printing pattern with progressively decreasing distances between the printed filaments, spanning from 1.2 to 0.1 mm (Figure 2B right). This test indicated a minimum required gap between filaments of 500 µm.

Finally, we performed a collapse test to examine the bioink stability against gravity. For this purpose, we used a fabricated construct with increasing distance ranging from 0.1 to 1.2 mm. The printed bioink showed no deflections in all filament distances up to 1.2 mm (Figure 2C).

We intended to print the hydrogel in three different macroporous mesostructures based on corresponding CAD designs (Figure 7), differing in filament diameter (D6 or D5) or pore size (P6 or P12) but with the same relative layer height of 75% of the filament diameter (H75). The samples D6P6H75 yielded pore sizes of 665 ± 308 µm, while the structures D6P12H75 yielded pore sizes of 910 ± 115 µm (Figure 2D). The samples D5P6H75 unfortunately did not yield consistent pore sizes throughout the printed construct, suggesting that the small needle diameter of 500 µm was not suitable for printing consistent macroporous mesostructures, including cells under the conditions used here. As a result, we performed biological analyses but excluded the D5P6H75 samples for mechanical analyses, as—due to the inhomogeneous pore sizes within the samples—it would not have been possible to draw conclusions regarding structure‐property relationships and their effects on the cells. Immediately after printing, all constructs were imaged to investigate the cells’ distribution. We conducted an initial examination using brightfield microscopy (Figure 2E), which offers a broad overview but does not capture the entire sample in detail. Consequently, we used fluorescent imaging to confirm that cells were equally distributed in the hydrogel (Figure 2F).

**FIGURE 3:**
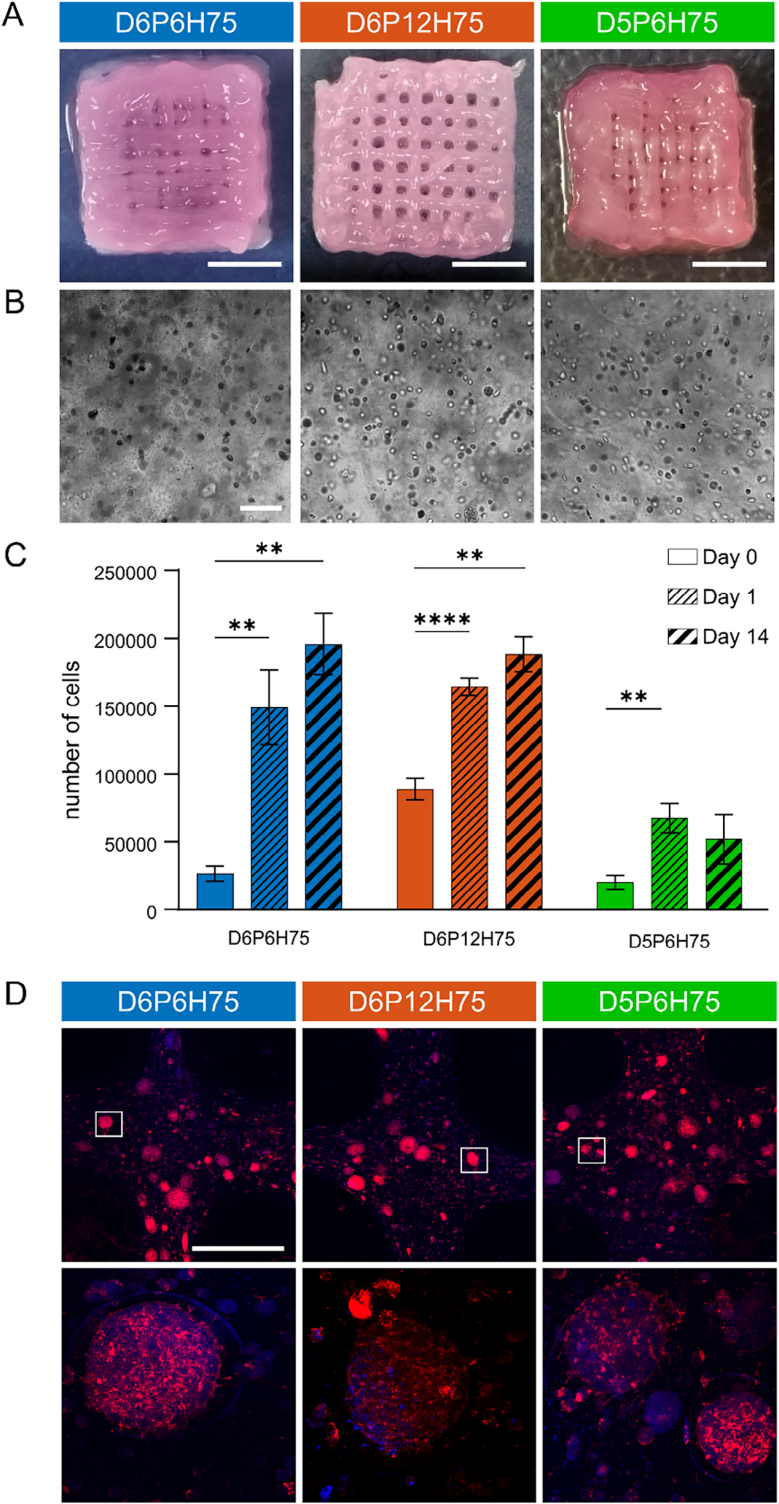
Macroporous mesostructures and cell behavior after 14 days under culture conditions. (A) Representative pictures of cell‐laden printed constructs with different macroporous mesostructures, D6P6H75, D6P12H75, and D5P6H75. Scale bar 5 mm. (B) Corresponding brightfield images of U87 cells after 14 days in the hydrogel. Scale bar 100 µm. (C) Metabolic activity was tested using 5 mm punches with Presto Blue HS at day 0, 1, and 14. The number of cells was achieved by correlating the sample absorbance to a conducted standard absorbance curve created via measuring known cell numbers for each individual experiment. Statistical significances were calculated using two‐way ANOVA followed by Tukey–Kramer tests for multiple comparisons. Significance values: ^**^ *p* < 0.01; ^****^ *p* < 0.0001. (D) Fluorescent tdTomato (red) transduced U87 cells embedded in the hydrogel (day 14). Nuclei were stained with DAPI (blue). Scale bar 500 µm. Magnifications of cell clusters (bottom row).

The printed constructs remained structurally stable under culture conditions for up to 14 days, as shown in Figure 3A. Brightfield microscopy (Figure 3B) was used to assess cell distribution within the constructs, while cell viability was evaluated through the metabolic assay PrestoBlue HS. This assay was performed immediately after printing, as well as after 1 and 14 days in culture. PrestoBlue HS was chosen as it resembles a non‐destructive method, enabling multiple readings of one sample over time. We report the measured metabolic activity in Figure 3C as an estimated cell number. This is based on a calibration curve generated by measuring the metabolic activity in reference samples with defined cell numbers. The metabolic activity in experimental samples was then compared to this reference to estimate cell counts. It is important to note that this method assumes a consistent relationship between metabolic activity and cells’ number. Therefore, if cells exhibit reduced metabolic activity, despite being alive, the assay may underestimate the actual number of viable cells present in the sample.

Under these considerations, our analyses show lower cell numbers per weight in the structures with smaller pore sizes (D6P6H75) than in those with larger pore sizes (D6P12H75) at day 0. We observed an increase in cell number over time, indicating cell proliferation. Significant differences occurred in all the structures between day 0 and day 1. Strikingly, D6P6H75 shows an increase in cell number, higher than what is explainable by their rate of proliferation possible within 1 day. This could be attributed to the small pore size, leading to less nutrient supply and therefore lower metabolic activity of the cells, indicated by lower cell numbers at day 0 compared to D6P12H75. We also observe this effect, although to a smaller extent, for macroporous mesostructure D5P6H75 with small pore sizes. Over time, this phenomenon seems to abate, as structures show reliable cell numbers at day 1, most probably due to the diffusion of nutrients into the hydrogel. Particularly in the structures D6P6H75 and D6P12H75, cell numbers also significantly increased between day 0 and day 14 (Figure 3C). The observation that cell numbers are lower in the macroporous mesostructure D5P6H75 and there is no increase in cell numbers from day 1 to day 14 could be attributed to the fact that no consistent macroporous mesostructure and thus macroporosity could be achieved in these structures. In addition, a possible explanation could be that due to the fusion of neighboring filaments, the cells did not have enough space to proliferate. This strengthens our decision to exclude this sample from the following mechanical analyses.

To investigate shape fidelity of the cell‐laden samples over time, we measured pore sizes again at day 14 and obtained 453.67 ± 134.25 and 778.67 ± 165.64 µm for D6P6H75 and D6P12H75, respectively. These results show that the pore size decreases over the course of 14 days in both structures, which is expected due to the overall shrinkage of the structures. This trend is slightly more pronounced in the structure with the smaller pores, D6P6H75, so that the difference in pore size between the two structures even increases compared to day 0. As a result, the fact that the mechanical response of both structures is similar on day 14 seems not to be related to changes in shape.

In general, our results confirm that cells are viable after the printing process and are able to proliferate up to 14 days. Cell behavior was monitored primarily through the assessment of metabolic activity. Nevertheless, we additionally captured individual images after 14 days (Figure 3D), showing that the cells tended to form clusters, likely as a result of proliferation. U87 glioblastoma cells often exhibit a tendency to form multicellular clusters or spheroid‐like aggregates within hydrogels. This reflects their inherent cell‐cell adhesion characteristics and can recapitulate aspects of tumor‐like organization in 3D culture [33, 34, 35].

### 2.2 Effect of Cells on Mechanical Properties of Printed Macroporous Mesostructures

**FIGURE 4:**
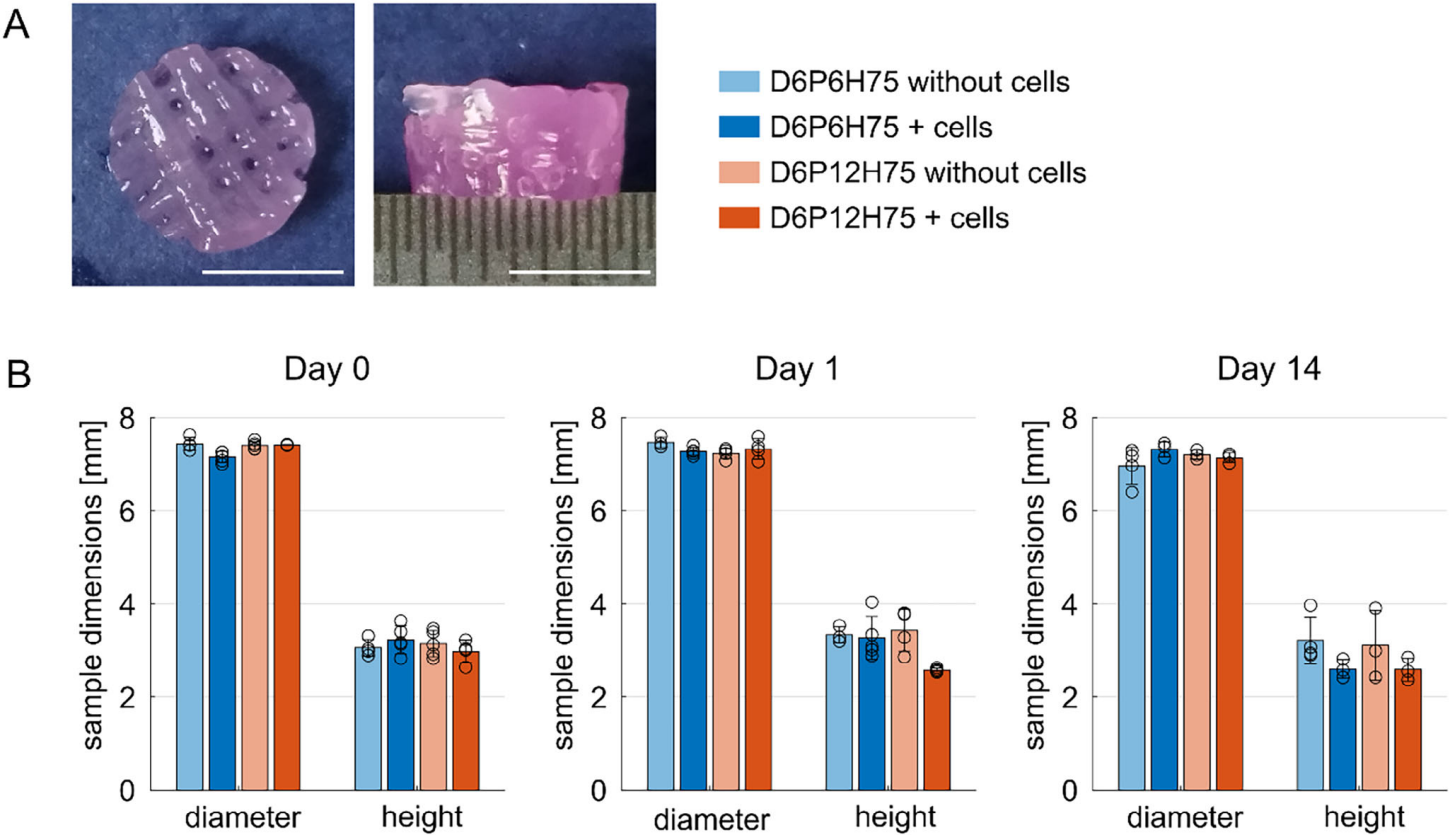
Dimensions of the samples for the mechanical tests. (A) Exemplary 8 mm sample side view (top) and top view (bottom) used for mechanical testing. Scale bars refer to 5 mm. (B) Overview of height and diameter for all printed ALG‐GEL samples at days 0,1, and 14.

To assess how the incorporation of cells into the printed macroporous mesostructures affects their nonlinear mechanical properties, we performed large‐strain cyclic compression‐tension as well as stress relaxation experiments. An exemplary image of a sample prepared for testing is shown in Figure 4A. Figure 4B shows an overview of the sample dimensions for samples mechanically tested at days 0, 1, and 14. The sample diameter was consistent for all samples, while the sample height varied slightly. Particularly on days 1 and 14, cell‐laden samples generally had slightly lower specimen heights than the structures without cells. However, samples on day 0 show, in general, a high structural integrity with sample heights closely matching 4 mm, which is the height of the designed CAD model. The correlation between sample height and sample diameter is plotted in Figure S1.

**FIGURE 5:**
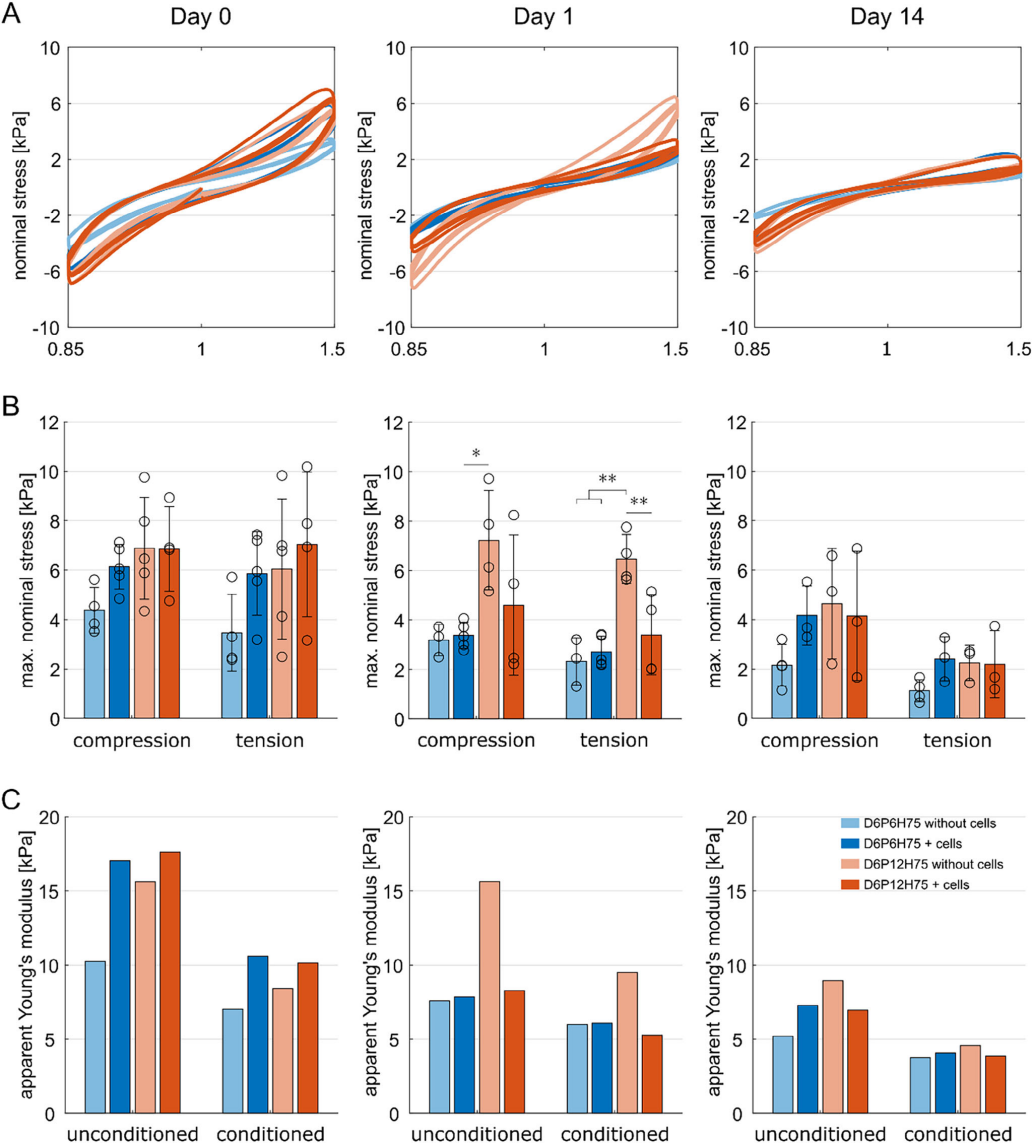
Mechanical analysis of printed macroporous mesostructures. (A) Cyclic compression‐tension behavior of 3D printed ALG‐GEL macroporous mesostructures up to a maximum strain of 15% on day 0 (left), 1 (center), and 14 (right). (B) Corresponding average maximum nominal stresses. (C) Corresponding apparent Young's moduli of the unconditioned and conditioned mechanical responses obtained from fitting the two‐term Ogden model to the first or third cycle, respectively, of cyclic compression‐tension data up to a maximum strain of 15%. The curves show the mean values, and the bars show the mean values with standard deviations. Significances were calculated using one‐way ANOVA, followed by Tukey–Kramer tests for multiple comparisons. Significance values: ^*^ *p* < 0.05, ^**^ *p* < 0.01.

Figure 5 shows the mechanical response during cyclic compression‐tension experiments for the two different macroporous mesostructures that showed a defined and consistent porosity, i.e., D6P6H75 and D6P12H75, with and without cells, respectively, at days 0, 1, and 14. All samples show similar mechanical characteristics (Figure 5A): nonlinearity (increasing stiffness for increasing strains), hysteresis (energy dissipation during each loading cycle represented by the area enclosed between the loading and unloading path), and conditioning (stiffer response during the first cycle than during subsequent cycles). Without cells, the macroporous mesostructure with a smaller pore size, D6P6H75, shows a softer mechanical response with lower maximum nominal stresses and lower apparent Young's moduli than the macroporous mesostructure with a larger pore size, D6P12H75 (Figure 5B, C; Figures S2 and S3, and Table S1). This observation could be attributed to the fact that the samples with smaller pores were generally less stable and more inhomogeneous than the samples with larger pores. The incorporation of cells led to an increase in stiffness on day 0. While this difference is clearly noticeable, especially for the smaller pore size (D6P6H75), it is not significant, which agrees with our previous findings [36] where only an amount of ≥6 000 000 cells mL^−1^ resulted in a significant change in the mechanical properties of molded samples. Interestingly, for the macroporous mesostructure with larger pore size (D6P12H75), this trend reverses starting at day 1 with lower maximum stresses and apparent Young's moduli for cell‐laden samples. While the macroporous mesostructures without cells show clear mechanical differences that are consistent over the investigated period of 14 days, all cell‐laden samples show a similar mechanical response, irrespective of the underlying macroporous mesostructure/print pattern. Our results thus suggest that the inclusion of cells equalizes the mechanical differences between macroporous mesostructures.

For cell‐laden samples, the stiffness drops notably between day 0 and day 1, but much less between day 1 and day 14. This observation could be related to the more significant change in cell numbers between day 0 and day 1 than between day 1 and day 14. It is important to note that only the alginate component of our bioink was crosslinked. The unbound gelatin polymers, which are not physically entrapped within the alginate crosslinked network, tend to dissolve under cell culture conditions, as previously reported [23, 37, 38]. Still, since all constructs were stored in cell culture conditions after their initial evaluation at day 0, gelatin release alone cannot explain the observed decrease in mechanical properties from day 0 to day 1 that only occurs for cell‐laden constructs. For samples without cells, on the contrary, there is only a minor change in the mechanical response between day 0 and day 1, but we see a notable drop in stiffness due to progressive degradation over time between day 1 and day 14. The cyclic torsional shear response shows similar trends to compression‐tension loading and is shown in Figure S4.

Overall, we may conclude that after the inclusion of cells, the macroporous mesostructure or macroporosity of samples is still relevant for the survival of cells but is less important for the overall mechanical response of the bioprinted construct, as effects induced by the cells predominate.

**FIGURE 6:**
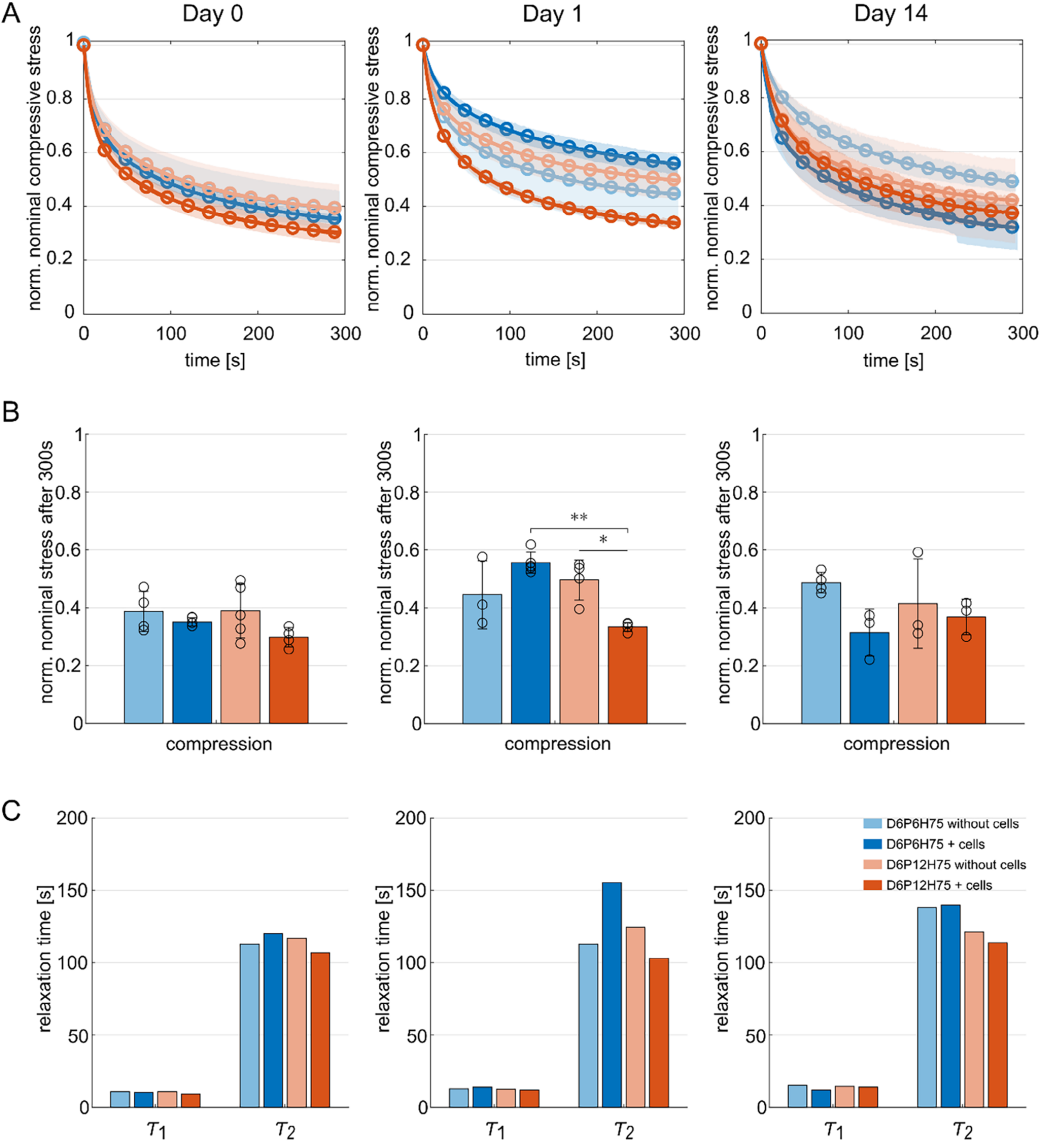
Normalized stress relaxation behavior in compression (solid lines) and two‐term Prony series fit (circles) of printed ALG‐GEL samples ionically crosslinked with 0:1 m CaCl_2_ on day 0, 1, and 14. (A) Average normalized stress relaxation behavior in compression at a maximum strain of 15%. (B) Normalized stress relaxation after 300s in compression. (C) Corresponding time constants τ_1_ and τ_2_ obtained from fitting a two‐term Prony series to the averaged stress relaxation curves in compression. The plots show the mean values with standard deviations. Significances were calculated using one‐way ANOVA, followed by Tukey–Kramer tests for multiple comparisons. Significance values: ^*^ *p* < 0.05, ^**^ *p* < 0.01.

Figure 6 shows the normalized stress relaxation behavior in compression for the two different macroporous mesostructures, i.e., D6P6H75 and D6P12H75, with and without cells, respectively, at days 0, 1, and 14. At day 0, all samples show a similar stress relaxation behavior (Figure 6A) with slightly lower normalized nominal stresses after a holding time of 300s for cell‐laden samples (Figure 6B). On day 1, the samples have become less viscous with a general trend of higher normalized nominal stresses after 300s. This trend is less pronounced for the cell‐laden macroporous mesostructure with larger pores, D6P12H75, which is significantly more viscous than its counterpart without cells and the cell‐laden macroporous mesostructure with smaller pores (D6P6H75).

The first and smaller relaxation times, represented by the first time constants τ_1_, are markedly consistent between the different samples and time points. The observed differences in stress relaxation behavior can thus be attributed to the differences in the second, higher relaxation times, i.e., the second time constants τ_2_ (Figure 6C). The stress relaxation behaviors in tension and torsional shear show similar trends to those in compression and are shown in Figures S5 and S6.

## 3 Conclusions

While different macroporous mesostructures printed in this study showed distinct mechanical properties without cells, this difference vanished after the incorporation of cells into the bioink. The cell‐laden constructs were stable over 14 days with decreasing stiffness over time. Interestingly, while the macroporous mesostructures without cells show similar properties on day 0 and day 1 (with only slight softening effects), but a significant decrease in stiffness between day 1 and day 14, cell‐laden macroporous mesostructures show a significant drop between day 0 and day 1 and only slight mechanical softening between day 1 and day 14. The cell proliferation was similar in both macroporous mesostructures, resulting in consistent pore sizes of 665 ± 308 and 910 ± 115 µm, respectively, which could be related to the fact that both structures showed similar macroscopic mechanical properties. The macroporous mesostructure with even smaller pores but problems due to fusion of neighboring strands are expectedly to result in inhibited cell proliferation. Our study highlights the importance of including cells when evaluating materials and structures for bioprinting and tissue engineering applications.

## 4 Experimental Section

### 4.1 Hydrogel Preparation

The hydrogel used was composed of gelatin (type A, 300 bloom derived from porcine skin, Sigma–Aldrich, Germany) and Sodium alginate type PH163 S2 (Vivapharm, JRS PHARMA GmbH & Co. KG) and prepared as follows. 5% (w/v) gelatin was first dissolved in Dulbecco's Phosphate Buffered Saline (DPBS, ThermoFisher, Invitrogen, Germany) on a rotational shaker at 37°C for 1 h. Subsequently, 2% (w/v) Sodium alginate type PH163 was added to the gelatin solution. The alginate‐gelatin hydrogel was prepared 12 h before printing and stored at 37°C. This ensured that bubbles were avoided in the final solution and that all the samples could be printed the same day with the same cell batch to reduce sample‐to‐sample variation.

### 4.2 Sample Design and Bioprinting

**FIGURE 7:**
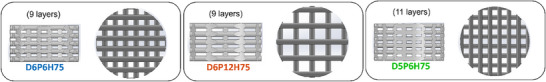
CAD design of the three macroporous mesostructures with different pore sizes and filament diameters. From left to right, the pore sizes are 600, 1200 and 600 µm, respectively, and filament diameters are 600, 600, and 500 µm, respectively. The layer height is 75% of the filament diameter for all samples.

We used the same design models and printing files as introduced in our previous study [16], but limited our current study to three macroporous mesostructures, which had shown significant differences in their mechanical properties before. We denote the designs differing in pore size and filament diameter by DxPyH75, referring to a nominal filament diameter and pore size of x and y hundred micrometers, respectively, and a layer height of 75% of the filament diameter (H75). Specifically, we printed the structures D6P6H75 (600 µm filament diameter, 600 µm pore size), D6P12H75 (600 µm filament diameter, 1200 µm pore size), and D5P6H75 (500 µm filament diameter, 600 µm pore size). We used the BioX bioprinter (BICO, Sweden) and set the printing parameters, e.g., temperature, pressure, speed, and layer height. We prepared macroporous samples by fabricating larger constructs and extracting cylindrical shapes using a surgical punch (Stiefel, Dublin, Ireland) with a diameter of 8 mm (see Figure 7). This way, the boundary effects were eliminated during subsequent mechanical testing. For cell printing, U87 cells were loaded in the bioink at a concentration of 2 000 000 cells mL^−1^. After 5 min at 4°C, the cartridge was incubated at 27°C for 1 h in the BioX temperature‐controlled print head. Finally, the samples were crosslinked using 0.1 m CaCl_2_ for 10 min and washed with Hanks' Balanced Salt Solution (HBSS, ThermoFisher, Invitrogen, Germany).

### 4.3 Printability Tests

To examine the proper gelation of the bioink, we followed the method that we previously described [16]. The bioink was first extruded at the required pressure to have a continuous flow to observe the bioink's extrudability. Then, we measured the printability value (*P_r_*) of the bioink by printing two layers of crossed patterns, using the formula:

$$P_{r}=\frac{L^{2}}{16A},$$

where L is the perimeter and A is the area. For an ideal gelation condition or perfect printability, the interconnected channels of the constructs would have a square shape with a *P_r_* value of 1. Larger *P_r_* values indicate a higher gelation degree of the bioink, smaller *P_r_* values indicate a lower gelation degree. A *P_r_* value close to 1 means appropriate gelation and high printability of the bioink [16, 17]. To calculate the *P_r_* value, we captured top‐view images of the samples and determined the perimeter and area of interconnected channels. Then, we analyzed these channels using the ImageJ software (n = 5). Afterward, we determined the minimum required gap between two adjacent filaments by the fusion test. Briefly, we designed and printed a pattern with different gaps, and after capturing optical images, we determined the minimum gap for printing without any filament fusion. In addition, we utilized the collapse test to measure the resistance of the bioink against the gravitational force. We printed the bioink on an artifact resembling the possible gaps between two printed filaments.

### 4.4 Mechanical Measurements

For mechanical testing, cyclic and stress relaxation tests in compression, tension, and torsional shear were performed using a Discovery HR‐3 rheometer (TA instruments, New Castle, Delaware, USA) equipped with an 8 mm diameter parallel geometry, as described in detail in our previous study [16]. To glue the samples to the upper and lower geometries of the rheometer, 8 mm circular pieces of fine sandpaper with a grain size of 180 µm were glued to the top (upper loading surface) geometry and the bottom heat plate (lower loading surface). The sample was then attached to the two sandpaper pieces using Pattex superglue ultra gel (Henkel AG & Co. KGaA, Düsseldorf, Germany).

### 4.5 Hyperelastic Properties

To quantify the complex elastic, time‐independent mechanical properties of 3D printed ALG‐GEL macroporous mesostructures, characterized by pronounced nonlinearity and compression‐tension asymmetry, we use the theory of nonlinear continuum mechanics and postulate the existence of a strain‐energy function 𝛹. We decompose the strain‐energy function 𝛹 into an isochoric and a volumetric part 𝛹 = 𝛹_iso_ + 𝛹_vol_.

Based on our previous studies on alginate‐based hydrogels [39, 40], in which a pronounced (S‐shaped) nonlinearity and a higher resistance of hydrogels in compression than in tension at the same strain (compression‐tension asymmetry) could be experimentally observed, we use the two‐term Ogden model [41] for the isochoric part with its phenomenological, isotropic hyperelastic strain‐energy function

$$\psi_{\mathrm{iso}}^{O}=\frac{\mu_{1}}{\alpha_{1}}\left[{\bar{\lambda }}_{1}^{\alpha_{1}}+{\bar{\lambda }}_{2}^{\alpha_{1}}+{\bar{\lambda }}_{3}^{\alpha_{1}}-3\right]+\frac{\mu_{2}}{\alpha_{2}}\left[{\bar{\lambda }}_{1}^{\alpha_{2}}+{\bar{\lambda }}_{2}^{\alpha_{2}}+{\bar{\lambda }}_{3}^{\alpha_{2}}-3\right]$$

expressed in terms of the nonlinearity parameters α_1_and α_2_, the shear moduli μ_1_and μ_2_, and the isochoric principal stretches ${\bar{\lambda }}_{i}=J^{-1/3}\lambda_{i}$, which were a function of the Jacobian *J* and the principal stretches λ_*i*_.

For the volumetric part, we consider the generalized strain‐energy function proposed by Ogden [41] with an empirical coefficient of −2 [42, 43].

$$\psi_{\mathrm{vol}}^{O}=\frac{\kappa }{4}\left(-2\ln J+J^{2}-1\right)$$

where κ was the bulk modulus and *J* the Jacobian. Since our mechanical test setup does not contain information about the volumetric deformation of the sample, we evaluate the bulk modulus

$$\kappa =\frac{2\mu \left(1+\nu \right)}{3\left(1-2\nu \right)}$$

in terms of the classical shear modulus μ and the initial Poisson's ratio ν. Furthermore, we use the relation $\mu =\frac{1}{2}\sum_{i=1}^{n}\mu_{i}\alpha_{i}=\frac{\mu_{1}\alpha_{1}}{2}+\frac{\mu_{2}\alpha_{2}}{2}$ from the linear regime [44] for the two‐term Ogden model and determine the (apparent) Young's modulus *E* from the classical shear modulus μ and the Poisson's ratio υ as *E*  =  2μ(1 + *v*). Under the assumption of incompressibility for hydrogels due to their high water content (ν = 0.5) [36], we obtain *E* = 3μ.

In the experiment, however, (large) deformations with certain inhomogeneities due to gluing of the individual samples to the upper and lower sample holder occur (Figure S2), and the actual boundary conditions need to be captured through finite element simulations [43, 45]. To avoid numerical issues, we assume near‐incompressibility for our finite element simulations and set ν = 0.49. Finally, we determine the optimal set of material parameters for the two‐term Ogden model by coupling our simulation model with an optimization routine [43].

### 4.6 Viscoelastic Properties

To characterize the time‐dependent mechanical properties of 3D printed ALG‐GEL macroporous mesostructures during stress relaxation in compression, tension, and torsional shear, we apply a two‐term Prony series.

$$\sigma \left(t\right)=\sigma_{\infty }+\sum_{i=1}^{n}\left[\sigma_{i}-\sigma_{\infty }\right]e^{-\frac{t}{\tau_{i}}}$$

with the plateau stress σ_∞_, the peak stress σ_*i*_, and two characteristic time constants τ_1_ and τ_2_. While the first time constant τ_1_ captures the relaxation response on short time scales, τ_2_ describes the behavior on long time scales. In accordance with our previous studies on alginate‐based hydrogels [39, 40], we could confirm that a two‐term Prony series satisfactorily captures the time‐dependent characteristics of 3D printed ALG‐GEL macroporous mesostructures.

### 4.7 Cell Culture and Cell Analysis

Cell maintenance: U87 cells (U‐87 MG, ATCC HTB‐14, LGC Standards GmbH, Germany) and TdTomato‐farnesyl expressing U87 reporter cells were cultured in Minimal Essential Medium (MEM) (21090‐055, Gibco, MA, USA) supplemented with 10% FCS (10270‐106 Life Technologies, MA, USA), 1% Penicillin (50U mL−1), Streptomycin (50 µg mL−1) (15140‐122 Life Technologies, MA, USA), 1% 200 mm GlutaMAX (35050‐038 Life Technologies, MA, USA), and 1% 100 mm sodium pyruvate (11360‐039 Life Technologies, MA, USA). The cell lines were split twice per week. TdTomato‐farnesyl expressing U87 reporter cells were generated as described elsewhere [46] and were used for fluorescent images.

### 4.8 Cell Viability

Samples were obtained by using a surgical punch (Stiefel, Dublin, Ireland) with a diameter of 5 mm to extract sections from the macroporous mesostructure. PrestoBlue HS Cell Viability Reagent (A13262, Invitrogen, Germany) was used according to the manufacturer's protocol. The number of cells was achieved by correlating the sample absorbance to a conducted standard absorbance curve created via measuring known cell numbers for each individual experiment. The absorbance intensity was measured at days 0, 1, and 14 using the CLARIOstar (BMG LABTECH) plate reader at the wavelength 570 nm (using 600 nm as a reference).

### 4.9 Confocal Microscopy and Image Acquisition

Overviews of macroporous mesostructures were acquired using a Zeiss Axio Imager 2 microscope (20× air objective. Enlargements of samples were imaged using an inverted Olympus IX81 microscope equipped with an Olympus FV1000 confocal laser scanning system, a FVD10 SPD spectral detector, and diode lasers of 405 nm (DAPI), 473 nm (Alexa488), 559 nm (Cy3), and 635 nm (Cy5) (Olympus, Tokyo, Japan). Images shown were acquired using an Olympus UPLSAPO 10× (air, numerical aperture 0.4) and UPlan SAPO 60x (oil, numerical aperture 1.35). All were processed using ImageJ/Fiji [47].

### 4.10 Statistical Analyses

Statistical analysis for mechanical experiments of 3D printed ALG‐GEL macroporous mesostructures was performed by one‐way ANOVA with Tukey‐Kramer post‐test using Statistics Toolbox in MATLAB version R2023b 23.2.0.2428915 (MathWorks, USA). A value lower than 0.05 was considered to be significant (^*^ *p* < 0.05, ^**^ *p* < 0.01, ^***^ *p* < 0.001, ^****^ *p* < 0.0001). To evaluate the capability of the two‐term Ogden model to capture the time‐independent response of 3D printed ALG‐GEL macroporous mesostructures, the quality of the fitting results was quantified in terms of differences in the root mean squared error (RMSE) and the coefficient of determination (*R^2^*) between experimental data and model output for compression and tension. Statistical analysis for biological experiments of 3D printed ALG‐GEL macroporous mesostructures was performed by two‐way ANOVA with Tukey‐Kramer post‐test using GraphPad Prism 8.3.0 (Graphpad Software, San Diego, CA, USA).

## Conflicts of Interest

The authors declare no conflicts of interest.

## Supporting information




**Supporting file**: mabi70073‐sup‐0001‐SuppMat.pdf

## Data Availability

The data that support the findings of this study are available from the corresponding author upon reasonable request.
