# Supplementary material for: Cell Behavior and Complex Mechanical Properties of 3D Printed Cell‐Laden Alginate‐Gelatin Macroporous Mesostructures
Source: Macromol Biosci. 2025 Sep 17;25(12):e00204. doi: 10.1002/mabi.202500204 (PMC12704233; doi:10.1002/mabi.202500204)
Supplement: Supplementary file 1 — Supporting file: mabi70073‐sup‐0001‐SuppMat.pdf [file MABI-25-e00204-s001.pdf]

Supporting Information

Cell behavior and complex mechanical properties of 3D printed cell-laden alginate-gelatin macroporous mesostructures

Nicoletta Murenu[1], Jessica Faber[2], Anahita Ahmadi Soufivand[2], Monika Buss[2], Natascha Schaefer[1,+,], Silvia Budday[2,+,*]*

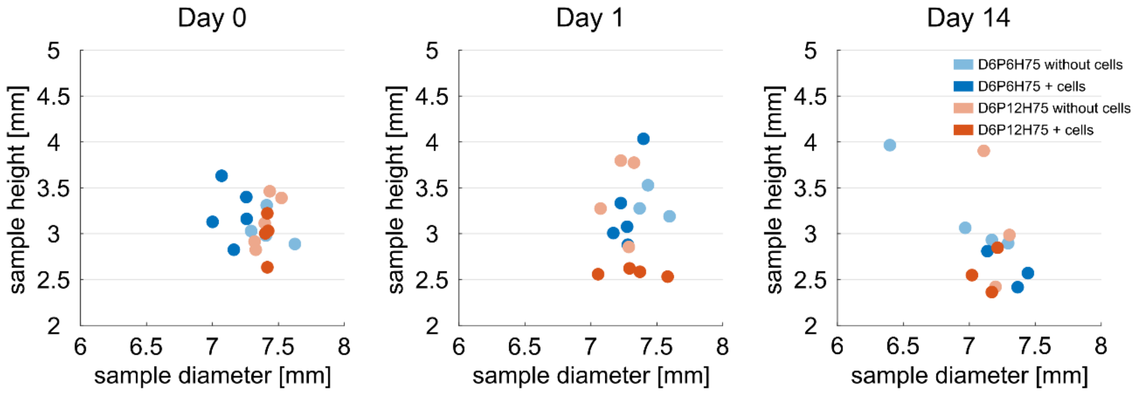

Figure S1. Correlation between the height and the diameter of the samples at day 1, 7 and 14 (light blue: D6P6H75 without cells, light blue: D6P6H75 with cells, light red: D6P12H75 without cells and dark red: D6P12H75 with cells).

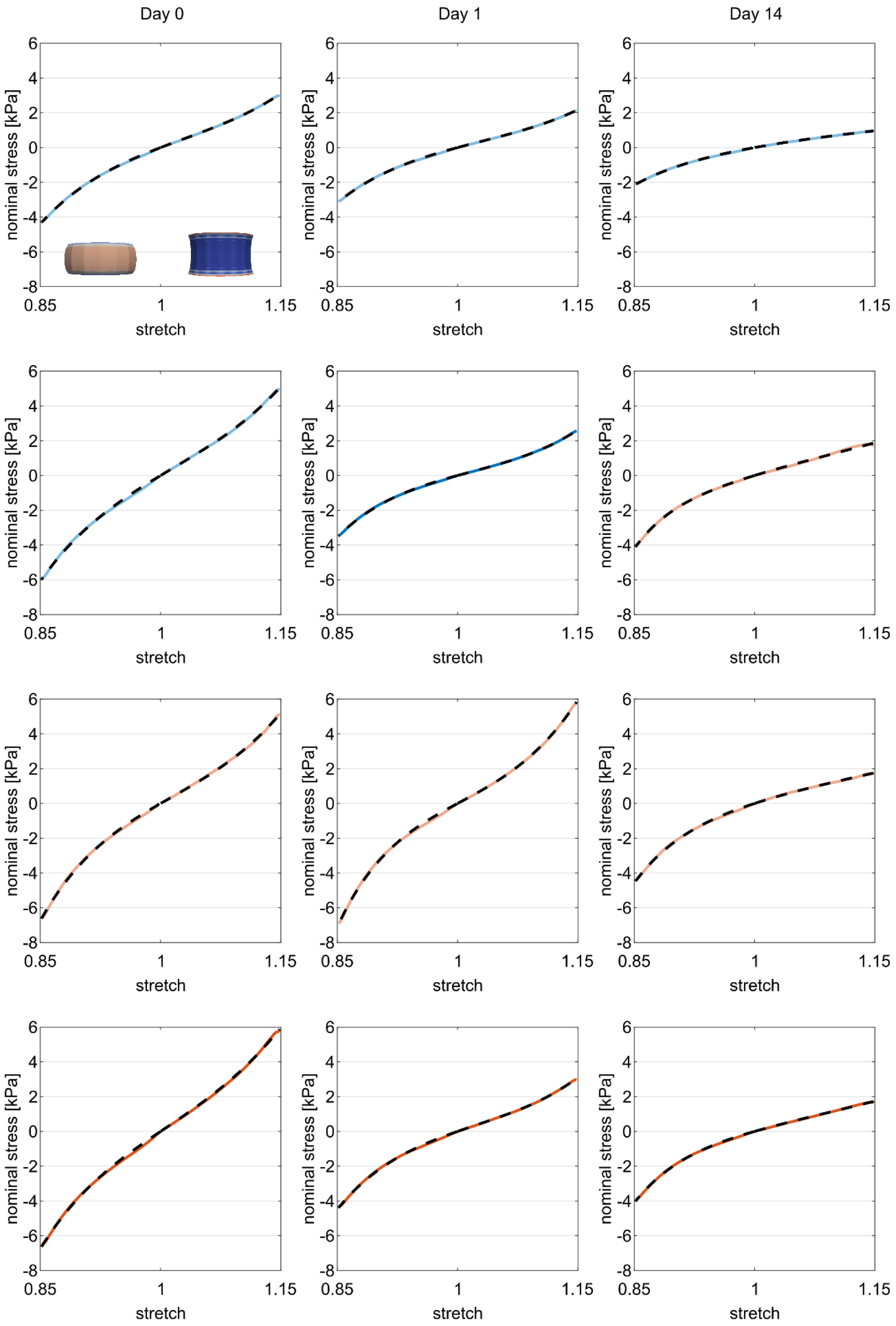

Figure S2. Two-term Ogden model calibrated with the average unconditioned mechanical response of 3D printed ALG-GEL macroporous mesostructures during the first cycle of cyclic compression-tension up to a maximum strain of 15% on day 0 (left), 1 (center), and 14 (right). The corresponding material parameters are listed in **Table S1**. The first plot includes finite

element simulations, which account for the inhomogeneous deformation and stress states during unconfined compression and tension of glued samples. The degrees of freedom of the bottom surface are fixed in space, while those of the upper surface are fixed in perpendicular directions to the applied load.

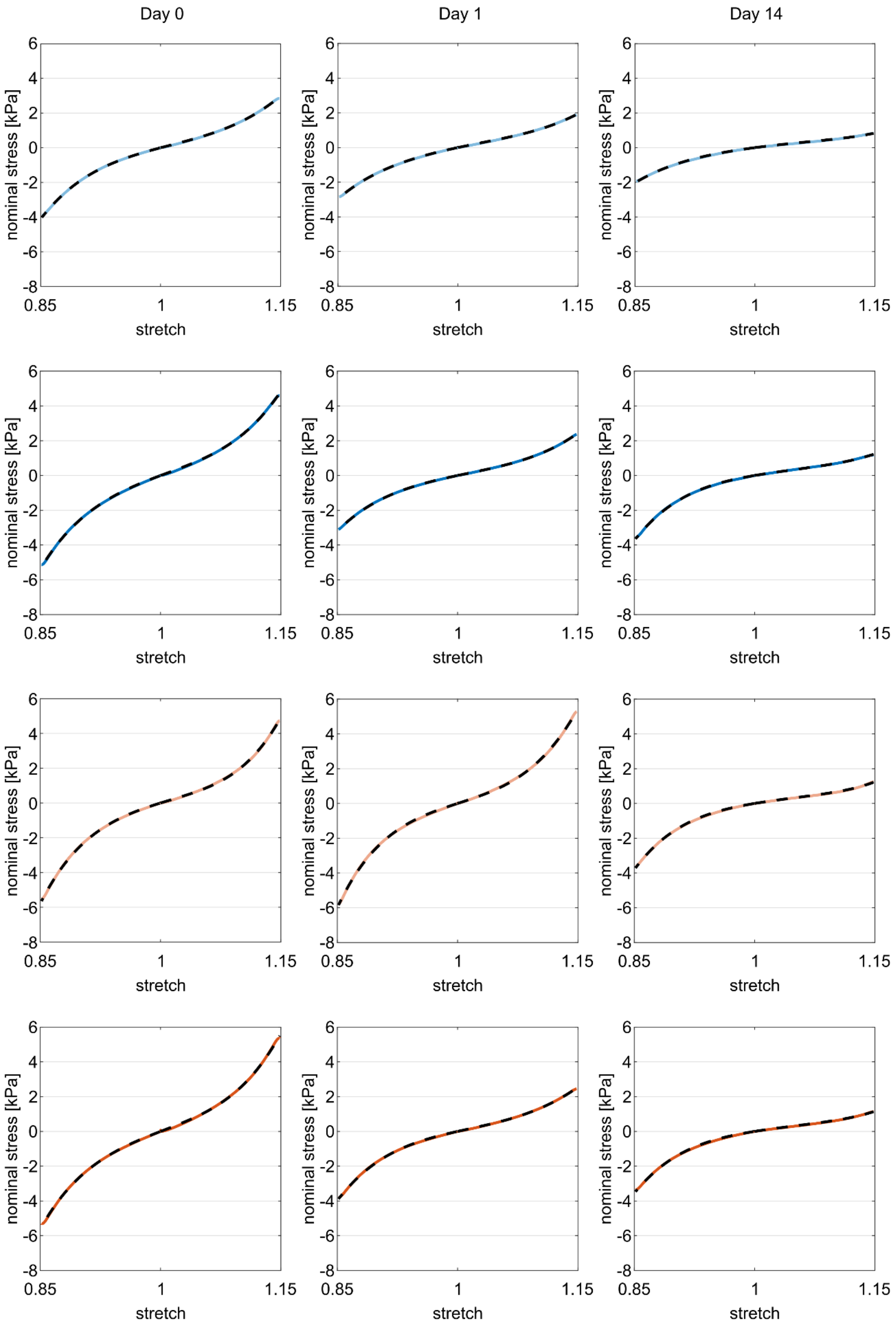

Figure S3. Two-term Ogden model calibrated with the average conditioned mechanical response of 3D printed ALG-GEL macroporous mesostructures during the third cycle of cyclic compression-tension up to a maximum strain of 15% on day 0 (left), 1 (center), and 14 (right). The corresponding material parameters are listed in **Table S1**.

**Label**	**two-term Ogden**								
	unconditioned								
	Day	α_1 [–]	α_2 [–]	μ_1 [Pa]	μ_2 [Pa]	RMSE [Pa]	R^2 [–]	μ [kPa]	app. YM [kPa]
D6P6H75 ctrl	D0	-3.03	14.17	-1593	141	22	1.00	3.41	10.23
	D1	-3.83	15.06	-976	88	18	1.00	2.53	7.59
	D14	-8.72	1.06	-137	2149	14	1.00	1.73	5.19
D6P6H75	D0	-1.00	12.89	-6723	359	59	1.00	5.68	17.04
	D1	-5.91	15.44	-543	131	18	1.00	2.62	7.86
	D14	-15.43	3.06	-39	1391	42	1.00	2.43	7.29
D6P12H75 ctrl	D0	-4.67	12.93	-1130	397	54	1.00	5.21	15.63
	D1	-8.72	13.95	-416	488	53	1.00	5.22	15.66
	D14	-10.97	1.00	-194	3847	34	1.00	2.99	8.97
D6P12H75	D0	-1.00	10.42	-3641	779	69	1.00	5.88	17.64
	D1	-3.37	16.40	-1227	84	31	1.00	2.76	8.28
	D14	-11.28	3.86	-135	805	23	1.00	2.32	6.96
	conditioned								
	Day	α_1 [–]	α_2 [–]	μ_1 [Pa]	μ_2 [Pa]	RMSE [Pa]	R^2 [–]	μ [kPa]	app. YM [kPa]
D6P6H75 ctrl	D0	-7.76	17.26	-363	108	16	1.00	2.34	7.02
	D1	-5.52	19.99	-598	35	11	1.00	2.00	6.00
	D14	-7.25	30.50	-341	1	16	1.00	1.25	3.75
D6P6H75	D0	-5.72	19.71	-776	134	26	1.00	3.54	10.62
	D1	-7.58	18.28	-337	82	8	1.00	2.02	6.06
	D14	-10.71	27.46	-247	2	15	1.00	1.35	4.05
D6P12H75 ctrl	D0	-8.09	25.55	-530	52	26	1.00	2.81	8.43
	D1	-11.10	20.33	-259	170	34	1.00	3.17	9.51
	D14	-11.40	33.45	-264	1	22	1.00	1.52	4.56
D6P12H75	D0	-3.21	21.15	-1342	116	35	1.00	3.38	10.14
	D1	-9.07	17.14	-238	80	17	1.00	1.76	5.28
	D14	-10.83	30.65	-236	1	23	1.00	1.29	3.87

Table S1. Material parameters, corresponding root mean square errors (RMSE) and coefficient of determination (R^2), classical shear moduli (μ), and apparent Young's moduli (app. YM) of the unconditioned and conditioned mechanical responses obtained from fitting the two-term Ogden model to the first or third cycle, respectively, of cyclic compression-tension data up to a maximum strain of 15% for printed ALG-GEL samples crosslinked with 0.1M $CaCl_2$.

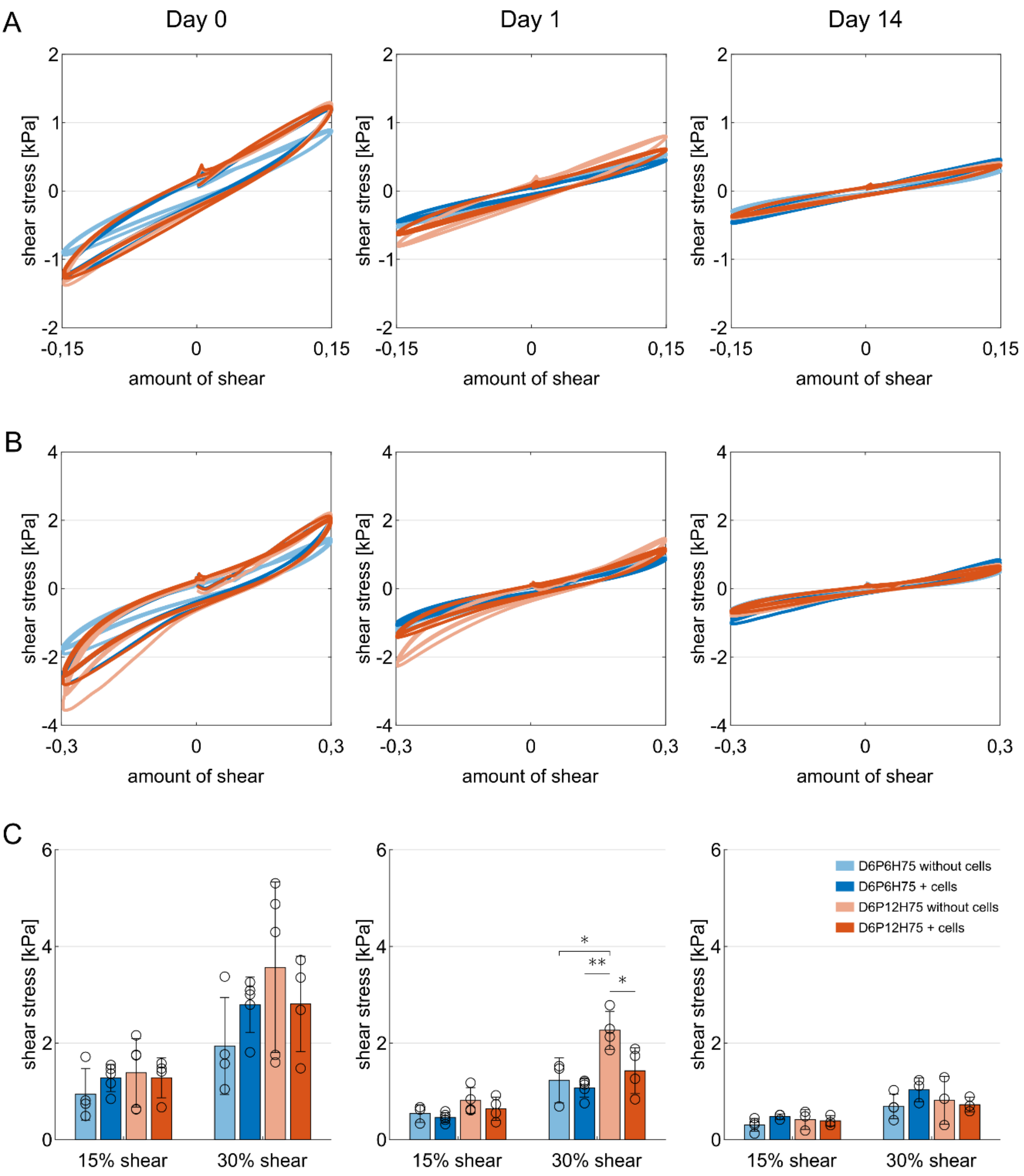

Figure S4. **A.** Cyclic torsional shear behavior of 3D printed ALG-GEL macroporous mesostructures up to a maximum shear of 15%, and **B.** 30% shear on day 0 (left), 1 (center), and 14 (right). **C.** Corresponding average maximum nominal stresses. Significances were calculated using one-way ANOVA followed by Tukey–Kramer tests for multiple comparisons. Significance values: *$p<0.05$, **$p<0.01$.

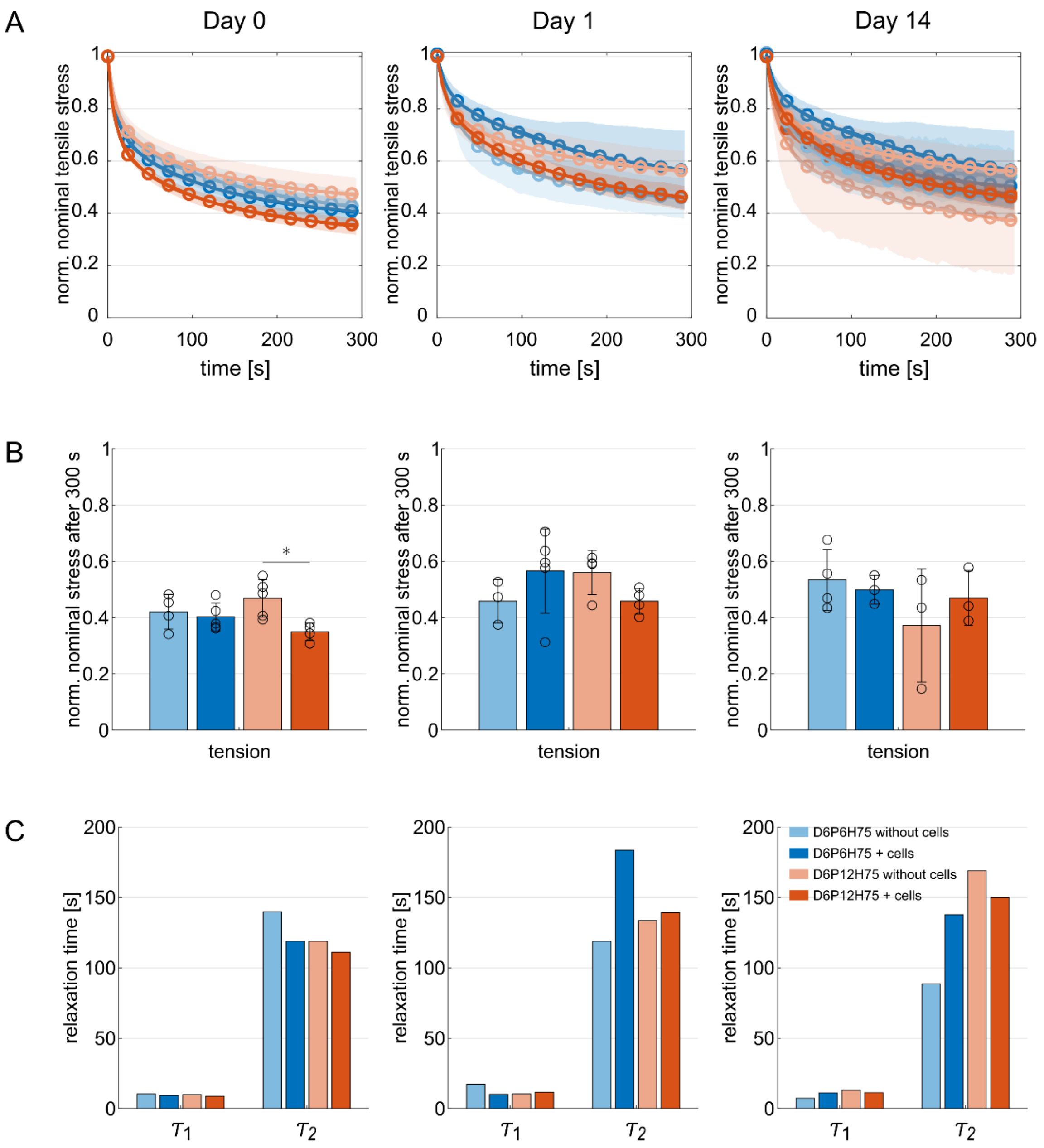

Figure S5. Normalized stress relaxation behavior in tension (solid lines) and two-term Prony series fit (circles) of 3D printed ALG-GEL macroporous mesostructures on day 0 (left), 1 (center), and 14 (right). **A.** Average normalized stress relaxation behavior in tension at a maximum strain of 15%. **B.** Normalized stress relaxation after 300s in tension. **C.** Corresponding time constants τ_1 and τ_2 obtained from fitting a two-term Prony series to the averaged stress relaxation curves in tension. The plots show the mean values with standard deviations. Significances were calculated using one-way ANOVA followed by Tukey–Kramer tests for multiple comparisons. Significance value: *$p<0.05$.

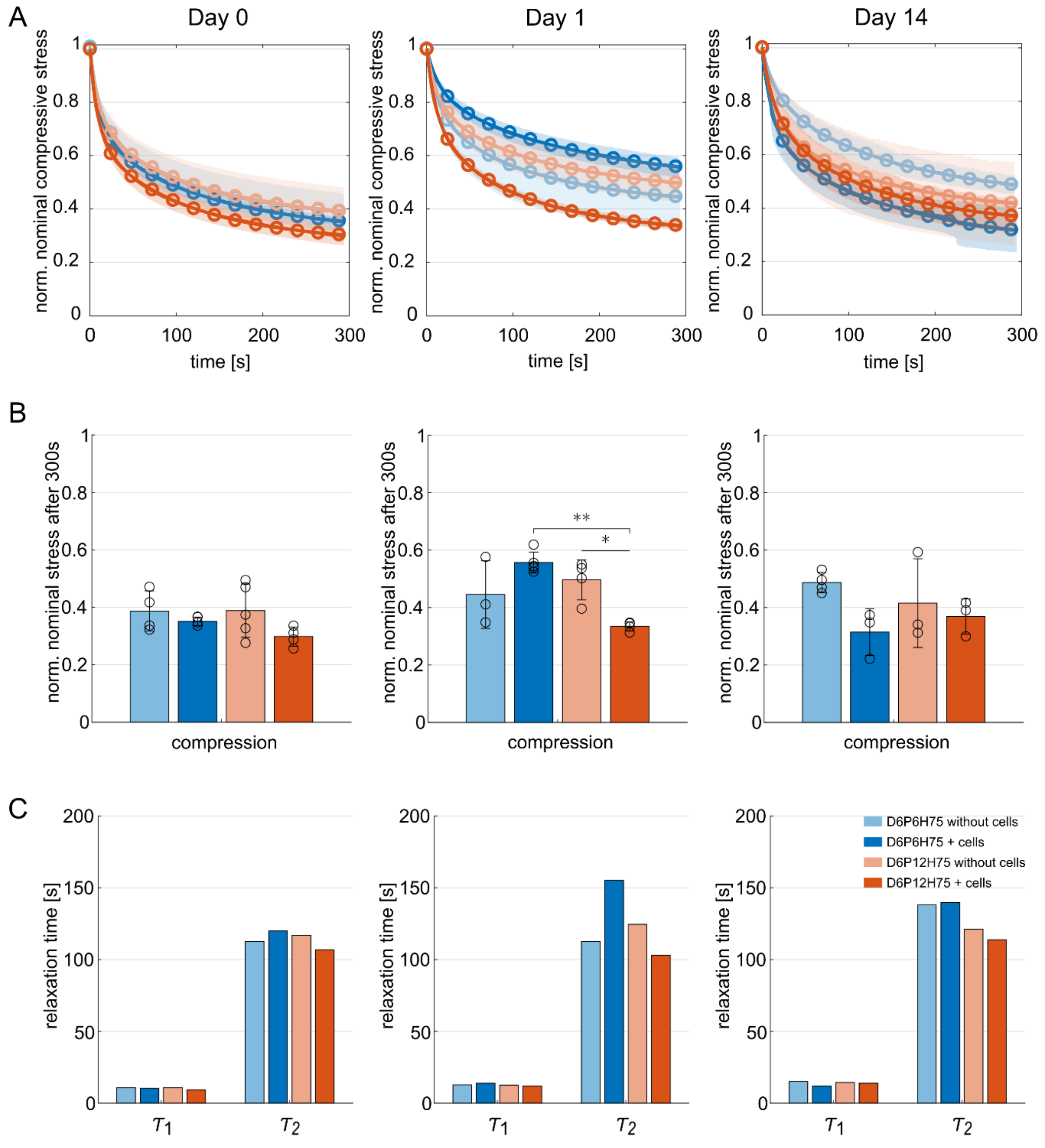

Figure S6. Normalized stress relaxation behavior in torsional shear (solid lines) and two-term Prony series fit (circles) of 3D printed ALG-GEL macroporous mesostructures on day 0 (left), 1 (center), and 14 (right). **A.** Average normalized stress relaxation behavior in torsional shear at a maximum shear of 30%. **B.** Normalized stress relaxation after 300s in torsional shear. **C.** Corresponding time constants τ_1 and τ_2 obtained from fitting a two-term Prony series to the averaged stress relaxation curves in torsional shear. The plots show the mean values with standard deviations. Significances were calculated using one-way ANOVA followed by Tukey–Kramer tests for multiple comparisons. Significance values: *p<0.05, **p<0.01, ***p<0.001.
